# Haemophagocytic lymphohistiocytosis driven by disseminated *Mycobacterium haemophilum* infection

**DOI:** 10.1093/skinhd/vzae020

**Published:** 2025-02-26

**Authors:** Parissa Irom, Ivan Rodriguez, Paige Kingston, Yasmin Gutierrez, Scott Worswick

**Affiliations:** University of Southern California, Los Angeles, CA, USA; Keck School of Medicine, University of Southern California, Los Angeles, CA, USA; Keck School of Medicine, University of Southern California, Los Angeles, CA, USA; Department of Dermatology, Keck School of Medicine, University of Southern California, Los Angeles, CA, USA; Department of Dermatology, Keck School of Medicine, University of Southern California, Los Angeles, CA, USA

## Abstract

*Mycobacterium haemophilum* is a nontuberculous mycobacteria that primarily affects immunocompromised patients. It can lead to a wide variety of clinical manifestations including infections of the skin, soft tissue and joints. Due to the significant heterogeneity in clinical presentation and difficulty isolating the organism, diagnosis can be difficult and is often delayed. Our patient’s course was further complicated by the development of haemophagocytic lymphohistiocytosis (HLH). Although *M. tuberculosis* infection is recognized as a potential association, HLH driven by a disseminated *M. haemophilum* infection has not yet been reported. Here we present a case of disseminated *M. haemophilum* infection in an immunocompromised patient who developed haemophagocytic lymphohistiocytosis.

## Teaser

*Mycobacterium haemophilum* is a non-tuberculous mycobacterium that mainly affects immunocompromised individuals, causing infections in the skin, soft tissues and joints. Diagnosing this organism is challenging due to its varied clinical symptoms and the difficulty in isolating it. In this case, an immunocompromised patient with a disseminated *M. haemophilum* infection also developed haemophagocytic lymphohistiocytosis.

**What’s already known about this topic?**

- Immunocompromised patients have an increased risk of infection and are thus susceptible to infections with atypical organisms.
- Haemophagocytic lymphohistiocytosis has been associated with *Mycobacterium tuberculosis* and some atypical mycobacteria ­infections.

**What does this study add?**

- A report of a patient with disseminated *Mycobacterium haemophilum* infection who developed haemophagocytic lymphohistiocytosis.

##  

Infection rates with nontuberculous mycobacteria (NTM) have steadily increased over the past several decades, predominantly affecting immunocompromised patients.^1,2^ NTM infections can cause a wide range of clinical presentations, ranging from localized cutaneous infection to disseminated disease.^2,3^  *Mycobacterium haemophilum* (*M. haemophilum*) is an NTM that commonly presents as asymptomatic papules and pustules, and progresses to painful ulcerations or necrotic abscesses.^2,4^ Less commonly, *M. haemophilum* can cause septic arthritis, osteomyelitis or disseminated disease.^4^

*Mycobacterium tuberculosis* and rarely NTM have been reported as causes of HLH; however, *M. haemophilum* as a cause of HLH has not yet been reported.^5^ HLH is a type of haemophagocytic syndrome (HPS), a life-threatening condition characterized by overstimulation of the immune system leading to systemic inflammation, hypercytokinaemia and multiorgan failure.^6^ Primary HPS is caused by genetic mutations, while secondary HPS results from a malignant, infectious or autoimmune stimulus.^7^ Here, we describe a case of an immunocompromised patient with disseminated *M. haemophilum* infection, with secondary HLH.

## Case report

A 30-year-old man with a history of immunoglobulin A nephropathy and allographic renal transplant secondary to end-stage renal disease, on tacrolimus, mycophenolate and low-dose prednisone, initially presented to an outside hospital for a rash present for several weeks, along with new-­onset cough, subjective fevers and crusting/weeping of his nose. He was also tachycardic and hypotensive. The patient complained of dizziness, fatigue, poor appetite and a rash.

After several days, he became confused and developed worsening renal function (creatinine increased to 5.2 mg dL^–1^ from his known baseline of 2.0 mg dL^–1^), uraemia and pancytopenia. An abdominal ultrasound demonstrated significant splenomegaly. Due to his rapid deterioration, he was initiated on cefepime and aciclovir and transferred to our institution for a higher level of care.

**Figure 1:**
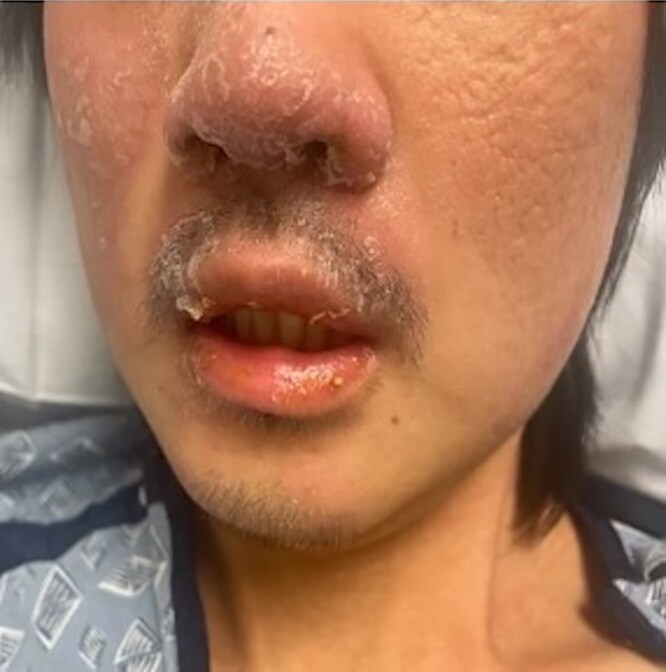
Scaly erythematous plaques on the right cheek, nose and upper lip.

**Figure 2:**
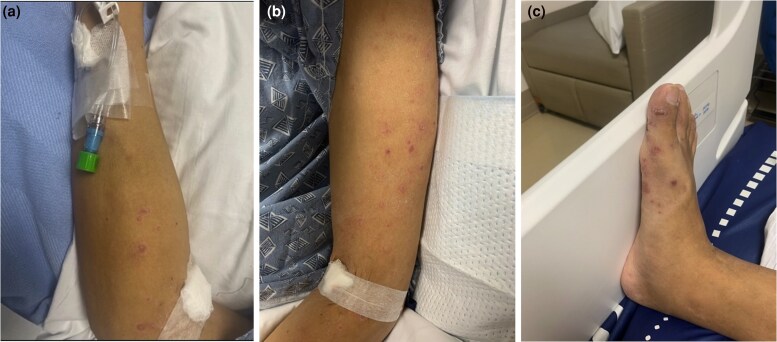
Representative photos of the patient's extremities. (a, b) Erythematous papules and scale on the upper left extremity and (c) right foot.

Upon arrival, his vital signs were stable. His confusion was persistent, and he was often unable to respond appropriately to questions. Skin exam was remarkable for erythematous, scaly plaques on the forehead, eyebrows, bilateral cheeks, nose and upper lip (Figure 1). He had scattered erythematous papules on the upper and lower extremities; notably, the trunk was clear (Figure 2).

**Figure 3:**
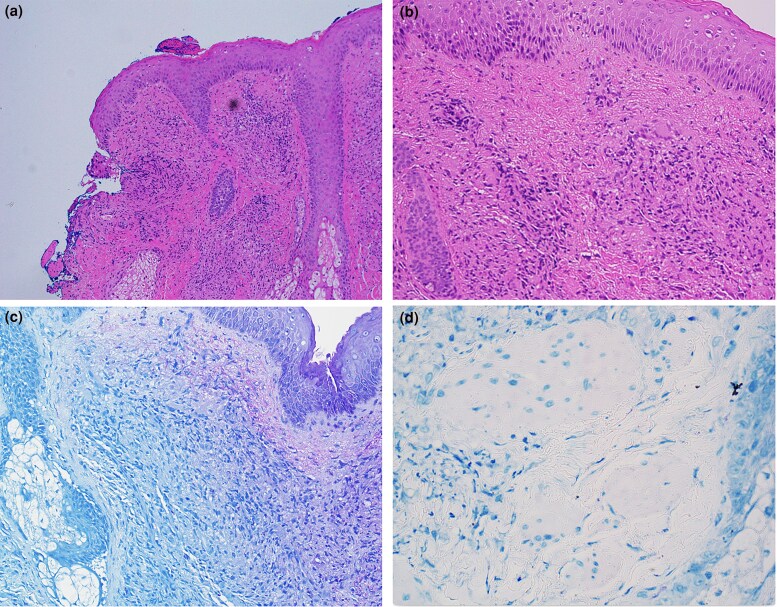
(a, b) Granulomatous dermatitis and chronic inflammation throughout the upper dermis with occasional giant cells and scattered acid-fast bacilli. The dermis shows fibrosis and reactive vascular ectasia. Haematoxylin and eosin, original magnification (a) ×10 and (b) ×20. (c, d) Acid-fast bacilli staining highlights small rod-shaped organisms consistent with mycobacteria. Fite, original magnification (c) ×20, and Kinyoun, original magnification (d) ×20.

Given the acute onset, there was significant concern for an infectious aetiology. Two punch biopsies of pustules on the left arm were sent for histopathology analysis and bacterial, acid-fast bacterial and fungal cultures (Figure 3). Empirical treatment with 100 mg doxycycline twice daily and 300 mg posaconazole twice daily was initiated. His mental status improved after 3 days of antimicrobial therapy. An infectious workup was performed and was remarkable for Epstein–Barr virus (EBV) and cytomegalovirus with subsequent polymerase chain reaction (PCR) positivity, although at low viraemic levels. Ganciclovir was added to the antimicrobial regimen initially; however, he only received one dose as it was recommended to be discontinued by the infectious disease team due to the low viraemia. Due to the acute and life-­threatening nature of his infections, his immunosuppressive regimen was decreased to only low-dose (2.5 mg q12h) ­tacrolimus.

Laboratory findings were remarkable for pancytopenia: white blood cell count was 2.18 × 10^9^ cells L^–1^, haemoglobin was 7.0 g dL^–1^ and platelets were 53 × 10^9^ cells L^–1^. He also had a hyperferritinaemia of 10 000 ng mL^–1^. Haematology was consulted and a work-up for suspected haemophagocytic lymphohistiocytosis (HLH) was performed. HLH workup was notable for a fibrinogen level of 152 mg dL^–1^, soluble interleukin-2 receptor level of 44 000 U mL^–1^ and a triglyceride level of 213 mg dL^–1^. He also had significant derangements in his hepatic panel: alkaline phosphatase was 456 U L^–1^, alanine transaminase was 89 U L^–1^ and aspartate transaminase was 150 U L^–1^. An abdominal ultrasound was performed and was notable for borderline hepatomegaly, thickening of the gallbladder wall and splenomegaly. Given the findings at this time, he was initially suspected to have HLH ­secondary to EBV.

Preliminary results of the punch biopsies from his left arm pustules demonstrated 3+ AFB organisms. *Mycobacterium tuberculosis* PCR was negative. After 2 weeks, the speciation of *M. haemophilum* was determined. Given these findings, the patient was diagnosed with HLH secondary to disseminated *M. haemophilum* infection. His antibiotic regimen was narrowed to ciprofloxacin, rifabutin and azithromycin, with plans for close follow-up by infectious disease specialists to continue until periodic cultures and clinical evaluation confirmed the infection had resolved. However, due to adverse effects suspected to be secondary to azithromycin, this was discontinued, and linzeolid was added. Regarding his immunosuppression regimen, he was to continue low-dose tacrolimus during his antibiotic treatment course, as advised by his transplant team. His skin lesions and laboratory findings gradually improved over 2 weeks of treatment. He was ultimately discharged with scheduled outpatient follow-up with dermatology, infectious disease and nephrology.

## Discussion

*Mycobacterium haemophilum* infections have been most commonly reported in immunosuppressed patients, such as transplant recipients.^3,4,8,9^ As a transplant recipient, our patient was at greater risk for *M. haemophilum* infection. In a 2020 study in Switzerland evaluating rates of infections 12-month status post-solid organ transplant (SOT) in 3541 patients, 55% of patients suffered 3520 infections, and of these, 63% of them were bacterial in origin.^10^ The most common SOT were kidney–pancreas (66%), kidney (66%), liver (59%), lung (58%) and heart (55%).^10^ While these rates are not specific for cutaneous infections, they provide a glimpse of bacterial infection rates when comparing types of SOT.

*Mycobacterium haemophilum* typically causes skin lesions that present as erythematous papules, plaques or nodules, which can progress to painful abscesses or ulcers.^4^ Such lesions are most frequently found on the extremities, especially over the joints, and less commonly on the trunk and face.^4^ While our patient presented with lesions on the upper and lower extremities consistent with typical manifestations of *M. haemophilum*, they also had erythematous plaques on the face. Facial involvement has been reported, Ishii *et al*. described a case of *M.haemophilum* infection mimicking leprosy in an immunocompromised woman.^11^ This case, along with ours, highlights the difficulty of differentiating *M. haemophilum* from other atypical causes of infection based on presentation.

Less commonly, *M. haemophilum* can lead to muscle or joint involvement.^4,12^ Greenwood *et al*. reported a case of an immunosuppressed patient who presented with pain in multiple joints, and red nodules near the affected joints.^12^ When immunocompromised patients present with joint pain, especially when cutaneous lesions are present, it is important to consider *M. haemophilum* and other NTM. Including acid-fast staining and *Mycobacterium* cultures in initial workups can lead to earlier diagnosis and can prevent the need for unnecessary surgical intervention, although it may be needed depending on the case.

Our patient was also diagnosed with HLH secondary to disseminated *M. haemophilum* infection. Although *M. tuberculosis* infection is recognized as a potential cause of HLH, NTM infection associated with HLH is uncommon.^5,13^ Notably, a 2023 literature review found 21 case reports of HLH associated with NTM infection.^5^ The majority of reported NTM-related cases of HPS were caused by disseminated NTM infection, where the lack of localized symptoms led to a delay in diagnosis.^7,13^ Interestingly, none of the cases was associated with *M. haemophilum* infection. Therefore, to our knowledge, this represents the first documented case of HLH associated with *M. haemophilum* infection.

Currently, there are no standard treatment guidelines for cutaneous *M. haemophilum* infection. Some studies suggest a multidrug regimen consisting of a macrolide, rifamycin and fluoroquinolones.^1^ Other suggested treatments include a combination of fluoroquinolones, rifampin and macrolides; or clarithromycin, ciprofloxacin and rifabutin, guided by susceptibility studies.^2,3^ There is one reported case of a patient responding well to 3 months of minocycline monotherapy.^3^ Duration, typically 3–12 months, and amount of therapy are dependent on the severity of disease presentation and the degree of immunosuppression in the patient.^1^ Our patient responded well to initial treatment with doxycycline and was transitioned to a regimen of rifampin, ciprofloxacin and linezolid, with the total length of therapy to be determined by close follow-up with infectious disease specialists.

Due to its lack of unified clinical manifestations, *M. haemophilum* infections may often be misdiagnosed. To achieve an accurate diagnosis, NTM infections should be considered in the differential diagnosis of cutaneous and subcutaneous conditions, particularly in immunocompromised patients. Further clinical and epidemiological research that advances our understanding of infections caused by atypical mycobacterium is needed.

## Data Availability

The data underlying this article will be shared on reasonable request to the corresponding author.
